# Supplementary material for: The combined HPV16-E2/E6/E7 T cell response in oropharyngeal cancer predicts superior survival
Source: Cell Rep Med. 2023 Nov 3;4(11):101262. doi: 10.1016/j.xcrm.2023.101262 (PMC10694628; doi:10.1016/j.xcrm.2023.101262)
Supplement: Document S1. Figures S1‒S3 and Tables S1 and S2 [file mmc1.pdf]

Cell Reports Medicine, Volume *4*

Supplemental information

The combined HPV16-E2/E6/E7 T cell response in oropharyngeal cancer predicts superior survival

Saskia J. Santegoets, Anouk Stolk, Marij J.P. Welters, and Sjoerd H. van der Burg

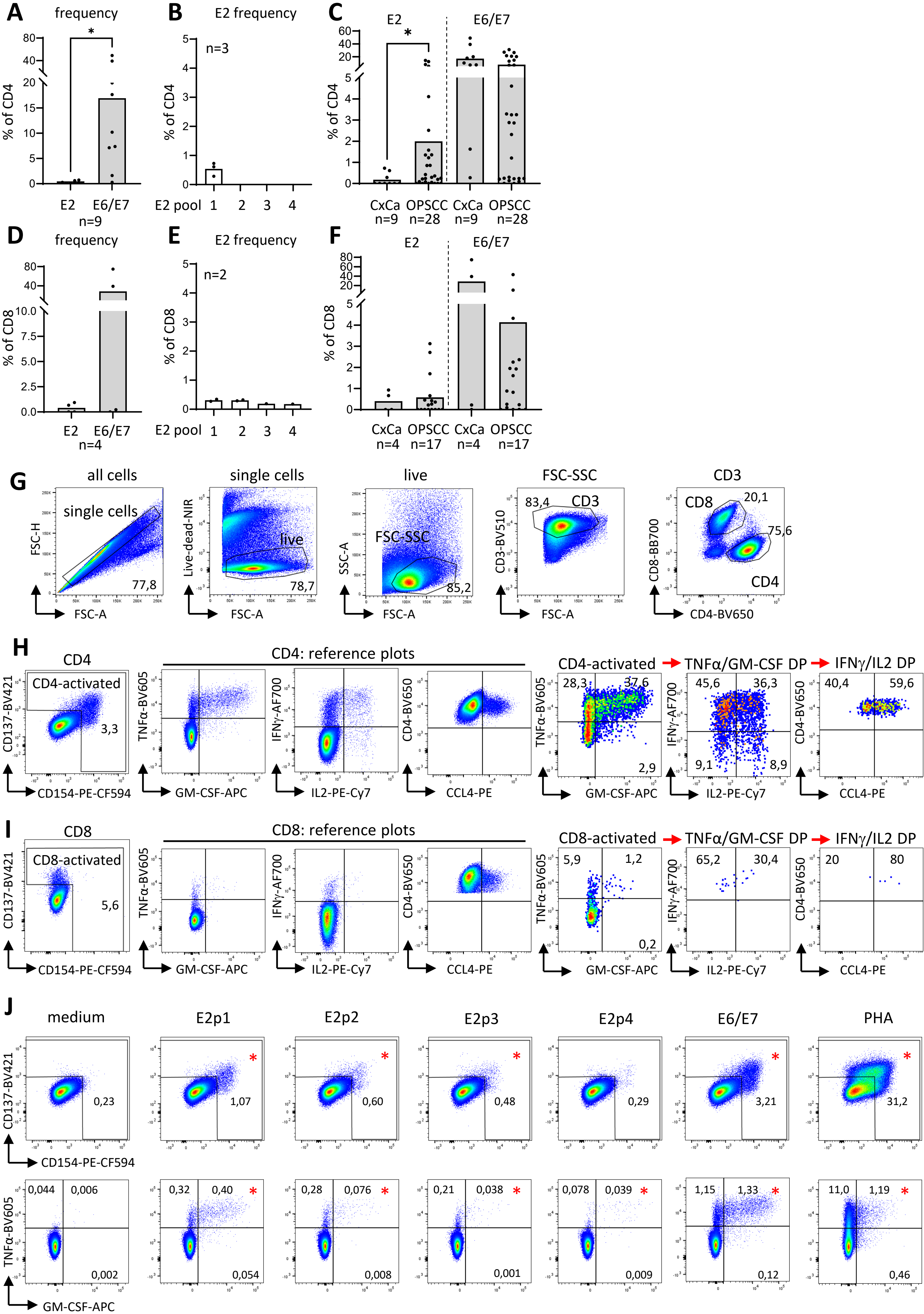
A
frequency
% of CD4
E2
E6/E7
n=9
B
E2 frequency
n=3
E2 pool 1 2 3 4
C
E2
E6/E7
CxCa n=9
OPSCC n=28
CxCa n=9
OPSCC n=28
D
frequency
% of CD8
E2
E6/E7
n=4
E
E2 frequency
n=2
E2 pool 1 2 3 4
F
E2
E6/E7
CxCa n=4
OPSCC n=17
CxCa n=4
OPSCC n=17
G
all cells
single cells
live
FSC-SSC
CD3
FSC-H
FSC-A
77,8
Live-dead-NIR
78,7
SSC-A
85,2
CD3-BV510
83,4
CD8-BB700
CD4-BV650
CD8
20,1
75,6
CD4
H
CD4
CD4-activated
3,3
CD137-BV421
CD154-PE-CF594
CD4: reference plots
TNFα-BV605
GM-CSF-APC
IFNγ-AF700
IL2-PE-Cy7
CD4-BV650
CCL4-PE
CD4-activated
TNFα/GM-CSF DP
IFNγ/IL2 DP
28,3
37,6
2,9
45,6
36,3
9,1
8,9
40,4
59,6
I
CD8
CD8-activated
5,6
CD8: reference plots
CD8-activated
TNFα/GM-CSF DP
IFNγ/IL2 DP
5,9
1,2
0,2
65,2
30,4
20
80
J
medium
E2p1
E2p2
E2p3
E2p4
E6/E7
PHA
0,23
1,07
0,60
0,48
0,29
3,21
31,2
0,044
0,006
0,002
0,32
0,40
0,054
0,28
0,076
0,008
0,21
0,038
0,001
0,078
0,039
0,009
1,15
1,33
0,12
11,0
1,19
0,46

Figure S1.

Figure S1. HPV16 E2-specific CD4+ and CD8+ T-cells can be found in TIL of CxCa patients, yet at lower frequency as E6 and E7-specific T-cells (related to Figure 1 and 2). E2 reactivity was determined in 9 CxCa patients with evident E6/E7 reactivity by manual gating. (A, D) Bar graph depicting total E2 and E6/E7-reactive CD4+ T-cells (A) and CD8+ T-cells (D). A response is depicted E2- or E6/E7-reactive when the percentage of cytokine-producing cells exceeds two times the medium control and has at least 10 positive spots in a gate. The total frequency of E2 and E6/E7-reactive T-cells is calculated as the SUM of all possible cytokine combinations as depicted in supplemental table I. (B, E) Bar graph depicting the frequency of the E2- or E6/E7-reactive CD4+ T-cells (B) and CD8+ T-cells (E) within E2 peptide pools 1 to 4. (C, F) Bar graph depicting frequency of E2- (left) and E6/E7-reactive (right) CD4+ T-cells (C) and CD8+ T-cells (F) in CxCa and OPSCC patients. To quantify polyfunctionality, HPV16-reactive CD4+ and CD8+ T-cells were subsequently subjected to manual gating. The gating strategy for polyfunctional T-cells is depicted for a representative OPSCC sample. (G) Cells were gated for singlets, live, CD3, CD4 and CD8 expression. (H, I) Activated CD4 (H) and CD8 (I) cells were selected by CD137 and/or CD154 expression and further analyzed for TNFα, GM-CSF, IFNγ, IL2 and CCL4 expression. Gates for cytokines were set on total CD4 and CD8 T-cells (reference plots). A gating example for sequential gating of the 5 cytokines is depicted for CD4 (H) and CD8 (I) cells. All possible cytokine combinations are given in supplemental Table I. (J) CD137/CD154 expression (top) and TNFα/GM-CSF expression (bottom) is depicted for CD4+ T cells in response to medium, E2p1-4 and E6/E7-loaded target cells and PHA. Red asterisk indicates a positive response, as defined by a frequency of at least two times the medium control.

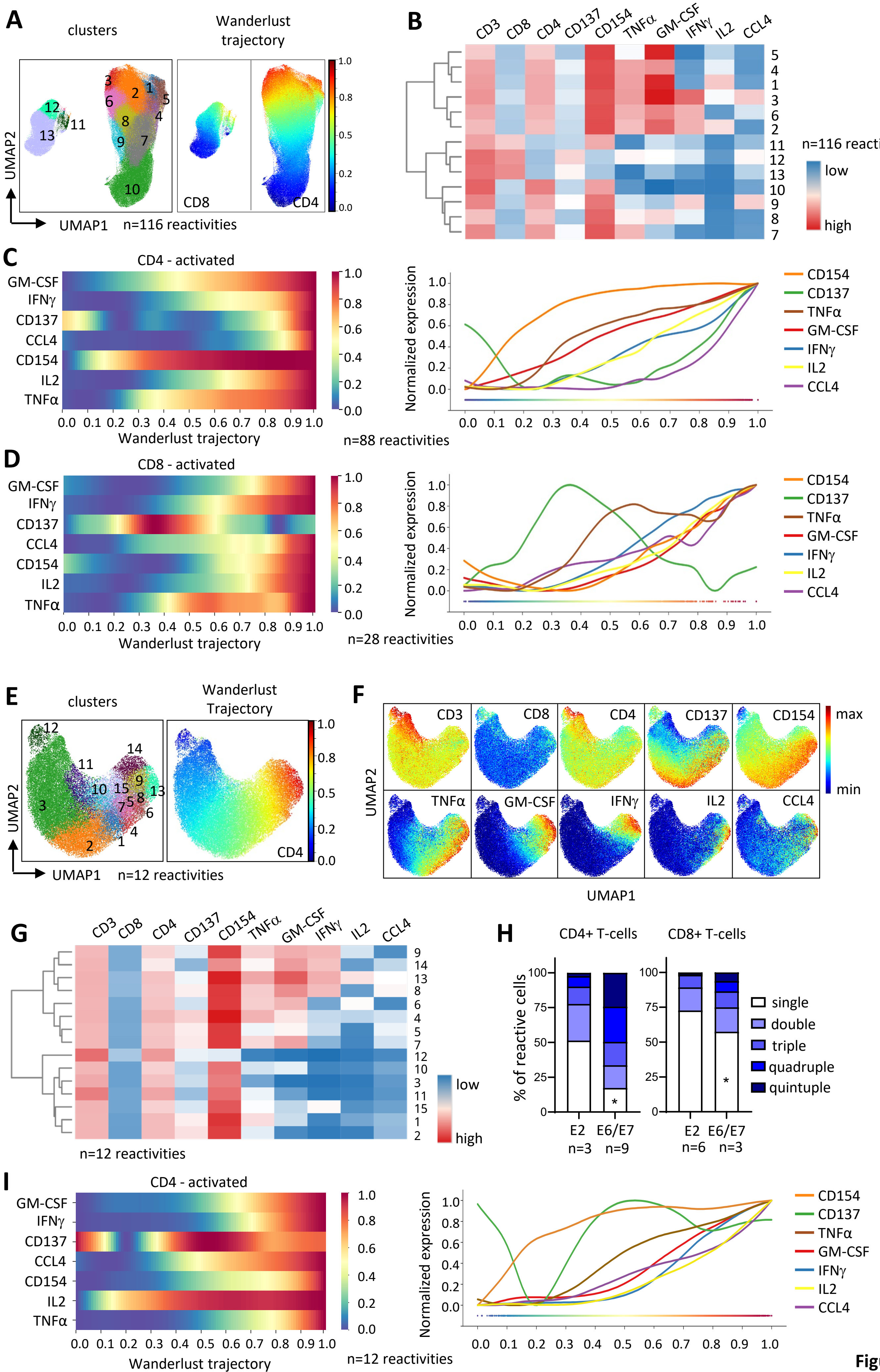
A
clusters
Wanderlust trajectory
UMAP2
UMAP1
n=116 reactivities
CD8
CD4
B
CD3 CD8 CD4 CD137 CD154 TNFα GM-CSF IFNγ IL2 CCL4
n=116 reactivities
low
high
C
CD4 - activated
GM-CSF
IFNγ
CD137
CCL4
CD154
IL2
TNFα
Wanderlust trajectory
n=88 reactivities
Normalized expression
CD154
CD137
TNFα
GM-CSF
IFNγ
IL2
CCL4
D
CD8 - activated
Wanderlust trajectory
n=28 reactivities
Normalized expression
E
clusters
Wanderlust Trajectory
UMAP2
UMAP1
n=12 reactivities
CD4
F
CD3
CD8
CD4
CD137
CD154
TNFα
GM-CSF
IFNγ
IL2
CCL4
max
min
UMAP2
UMAP1
G
CD3 CD8 CD4 CD137 CD154 TNFα GM-CSF IFNγ IL2 CCL4
low
high
n=12 reactivities
H
CD4+ T-cells
CD8+ T-cells
% of reactive cells
single
double
triple
quadruple
quintuple
E2 n=3
E6/E7 n=9
E2 n=6
E6/E7 n=3
I
CD4 - activated
Wanderlust trajectory
n=12 reactivities
Normalized expression

Figure S2.

Figure S2. HPV16 E2, E6 and E7-specific CD8+ and CD4+ T-cells in OPSCC and CxCa are polyfunctional (related to Figure 2). High-dimensional single cell data analysis of 116 activated CD4+ and activated CD8+ T-cell populations from OPSCC (A-D) and 12 activated CD4+ and activated CD8+ T-cell populations from CxCa (E-I) using OMIQ software. Dimensionality reduction using Uniform Manifold Approximation and Proximity (UMAP) analysis, FlowSOM consensus metaclustering and wanderlust trajectory analysis was performed on 245,072 activated CD4+ T-cells and 47,317 activated CD8+ T-cells for OPSCC and on 35,875 activated CD4+ T-cells for CxCa. (A) Overlay of 13 FlowSOM clusters (left) and Wanderlust trajectory (right; blue: begin and red: end) for the activated CD4+ and CD8+ T-cell populations in OPSCC plotted on the UMAP. (B) Hierarchically clustered heatmap of the FlowSOM clusters for OPSCC. Marker expression is shown as the z-score of median signal intensity per channel. (C, D) Heatmap plot (left) and line plot (right) displaying Wanderlust trajectory progression for 88 CD4-activated (C) and 28 CD8-activated (D) T-cell populations detected in OPSCC. (E) Overlay of 15 FlowSOM clusters (left) and wanderlust trajectory (right; color coding: blue: begin and red: end) plotted on the UMAP for the activated CD4+ T-cell populations in CxCa. (F) Expression intensity of the cell surface and cytokine markers in the CD4-activated T cells in CxCa plotted on the UMAP plot with blue as low and red as high expression. (G) Hierarchically clustered heatmap of phenotypes of the identified FlowSOM clusters is depicted. The indicated marker expression is shown as z-score of median signal intensity per channel. Blue: low expression, red: high expression. (H) Bar graphs depicting the percentage of single, double, triple, quadruple and quintuple cytokine-producing CD4+ T-cells (left) and CD8+ T-cells (right) for the total E2 and E6/E7-reactive T-cell populations in CxCa. (I) Heatmap plot (left) and line plot (right) displaying wanderlust trajectory progression for the markers CD137, CD154, TNFα, GM-CSF, IFNγ, IL-2 and CCL4 is depicted for CD4-activated T-cell populations detected in CxCa.

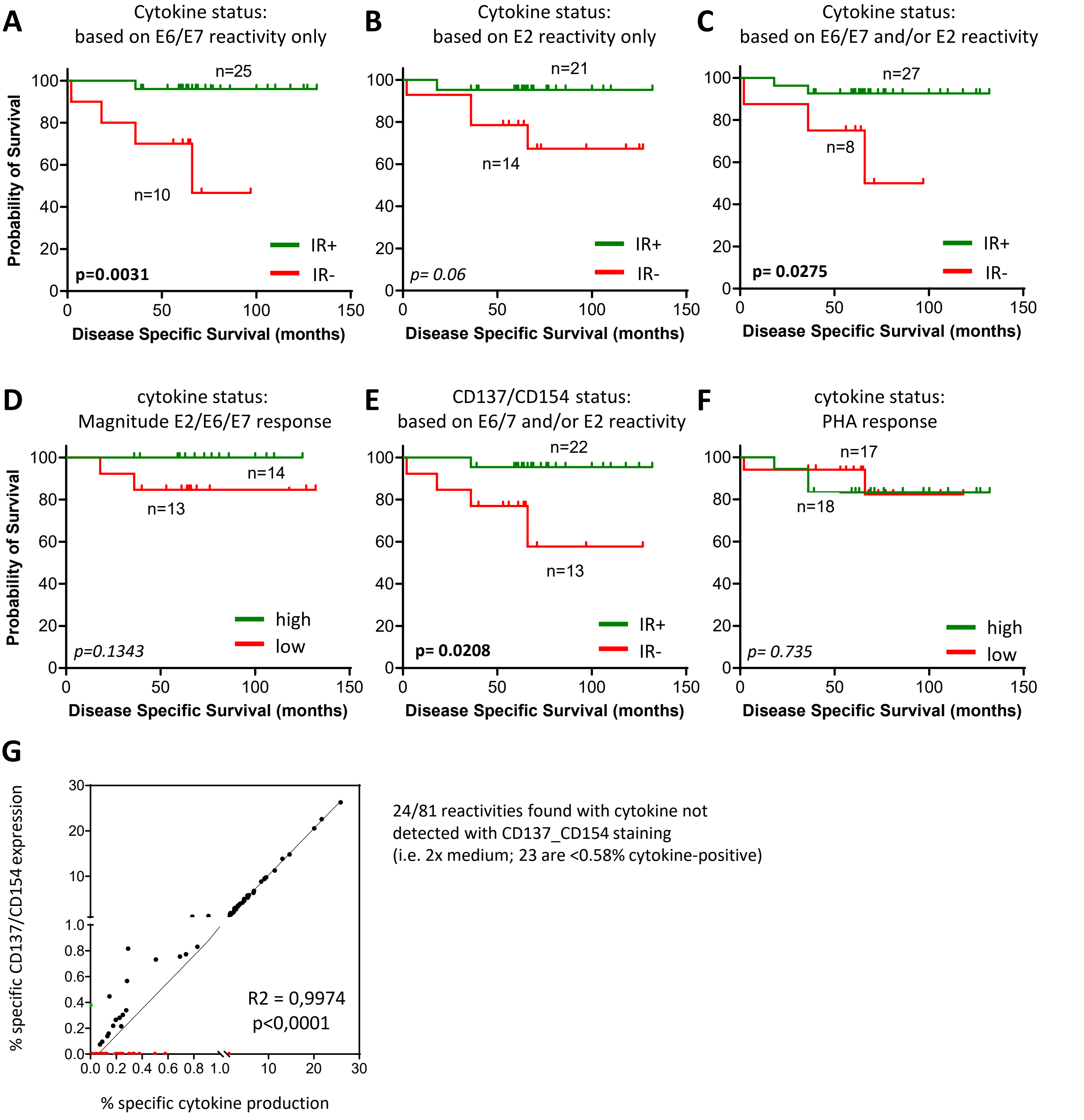

Figure S3. OPSCC patients with an immune response against HPV16 display superior survival (related to Figure 3). Kaplan-Meier survival curves of (A-C**)** 35 HPV16+ OPSCC patients who were analyzed by ICS. Patients were grouped based on the detection of an intratumoral HPV16 E2/E6/E7-specific T-cell response (i.e. immune response-positive (IR+; in green) or no detectable HPV16 E2/E6/E7-specific immune response (i.e. IR-negative (IR- in red)). IR status is given based on (A) detectable E6/E7-specific cytokine-producing T cells only, (B) E2-specific cytokine-producing T cells only and (C) E6/E7 and/or E2-specific cytokine-producing T cells for all patients. Kaplan-Meier survival curve of (D) 27 HPV16+IR+ OPSCC patients and (E, F) of 35 HPV16+ OPSCC patients who were analyzed by ICS. Patients were grouped based on (D) the detection of a high (i.e. > median (in green)) or low (i.e. < median (in red)) level of intratumoral total E2/E6/E7-specific cytokine-producing CD4+ T cells, (E) the detection of an intratumoral HPV16 E2/E6/E7-specific T-cell response (IR+; in green) or no detectable HPV16 E2/E6/E7-specific immune response (IR- in red) by CD137/CD154 expression and (F) the detection of a high (i.e. > median (in green)) or low (i.e. < median (in red)) level of intratumoral PHA-specific cytokine-producing CD4+ T cells.

(G) Correlation analysis between % specific CD137/CD154 expression and % specific cytokine production of E2 and E6/E7-specific CD4+ T cell populations for 81 different E2 and E6/E7-specific CD4+ T cell populations identified.

Table S1. Polyfunctional populations (Related to Figure 2 and STAR methods).

Population	How many cytokines
TNFa+GMCSF-IFNg+IL2-CCL4-	2
TNFa+GMCSF-IFNg+IL2-CCL4+	3
TNFa+GMCSF-IFNg+IL2+CCL4-	3
TNFa+GMCSF-IFNg+IL2+CCL4+	4
TNFa+GMCSF-IFNg-IL2+CCL4-	2
TNFa+GMCSF-IFNg-IL2+CCL4+	3
TNFa+GMCSF-IFNg-IL2-CCL4-	1
TNFa+GMCSF-IFNg-IL2-CCL4+	2
TNFa+GMCSF+IFNg+IL2-CCL4-	3
TNFa+GMCSF+IFNg+IL2-CCL4+	4
TNFa+GMCSF+IFNg+IL2+CCL4-	4
TNFa+GMCSF+IFNg+IL2+CCL4+	5
TNFa+GMCSF+IFNg-IL2+CCL4-	3
TNFa+GMCSF+IFNg-IL2+CCL4+	4
TNFa+GMCSF+IFNg-IL2-CCL4-	2
TNFa+GMCSF+IFNg-IL2-CCL4+	3
TNFa-GMCSF+IFNg+IL2-CCL4-	2
TNFa-GMCSF+IFNg+IL2-CCL4+	3
TNFa-GMCSF+IFNg+IL2+CCL4-	3
TNFa-GMCSF+IFNg+IL2+CCL4+	4
TNFa-GMCSF+IFNg-IL2+CCL4-	2
TNFa-GMCSF+IFNg-IL2+CCL4+	3
TNFa-GMCSF+IFNg-IL2-CCL4-	1
TNFa-GMCSF+IFNg-IL2-CCL4+	2
TNFa-GMCSF-IFNg+IL2-CCL4-	1
TNFa-GMCSF-IFNg+IL2-CCL4+	2
TNFa-GMCSF-IFNg+IL2+CCL4-	2
TNFa-GMCSF-IFNg+IL2+CCL4+	3
TNFa-GMCSF-IFNg-IL2+CCL4-	1
TNFa-GMCSF-IFNg-IL2+CCL4+	2
TNFa-GMCSF-IFNg-IL2-CCL4-	0
TNFa-GMCSF-IFNg-IL2-CCL4+	1

Table S2. Patient characteristics (Related to STAR methods).

**Patient ID** *	**Sex**	**Age****	**Tumor location**	**Received treatment**
**H35**	M	48	Tonsillar Fossa	RT
**H68**	F	64	Tonsil	RT
**H71**	F	62	Tongue base	RT
**H81**	M	48	Tonsillar Fossa	S+RT+CT
**H93**	M	57	Tongue base	S+RT
**H97**	M	70	Posterior wall	RT
**H103**	M	66	Tonsil	RT
**H104**	F	78	Tongue base	S+RT
**H133**	M	53	Tonsillar Fossa	RT
**H136**	F	47	Tonsil	CT+RT
**H138**	M	74	Tongue base	RT
**H139**	M	57	Tongue base	CT+RT
**H147**	M	57	Tonsil	CT+RT
**H148**	F	64	Tonsil	S+RT
**H149**	F	47	Tonsil	S+RT
**H150**	M	59	Tonsil	S+RT
**H160**	M	69	Tongue base	CT+RT
**H170**	M	60	Vallecula	S+RT
**H173**	F	60	Tongue base	CT+RT
**H180**	M	59	Tonsil	RT
**H182**	M	66	Tongue base	CT
**H185**	M	58	Tongue base	RT
**H188**	F	56	Tonsil	CT+RT
**H191**	M	39	Tonsil	RT
**H193**	M	67	Tongue base	RT
**H195**	F	62	Tongue base	CT+RT
**H196**	M	73	Tongue base	CT+RT+CX
**H202**	M	81	Tongue base	RT
**H206**	M	58	Tonsil	RT
**H208**	M	60	Tongue base	CT+RT
**H209**	M	70	Tonsil	CT+RT
**H211**	M	62	Tongue base	CT+RT
**H218**	M	58	Tongue base	RT
**H225**	M	67	Tongue base	RT
**H227**	F	44	Tonsil	CT+RT
**C176**	F	45	Cervix	S
**C178**	F	40	Cervix	S
**C194**	F	67	Cervix	S+RT
**C267**	F	49	Cervix	S+RT+CT
**C334**	F	42	Cervix	S+RT
**C446**	F	28	Cervix	S+RT
**C469**	F	42	Cervix	S
**C1016**	F	68	Cervix	S+RT
**C1051**	F	39	Cervix	S

* ‘H’ indicates OPSCC patients included in the P07-112 head and neck cancer study and “C” indicated CxCa patients included in the CIRCLE study.

** Age at diagnosis and sampling of tumor tissue pre-therapy

CT: chemotherapy; CX: cetuximab; F: female; M: male; RT: radiotherapy; S: surgical resection
