# Supplementary material for: The combined HPV16-E2/E6/E7 T cell response in oropharyngeal cancer predicts superior survival
Source: Cell Rep Med. 2023 Nov 3;4(11):101262. doi: 10.1016/j.xcrm.2023.101262 (PMC10694628; doi:10.1016/j.xcrm.2023.101262)
Supplement: Document S2. Article plus supplemental information [file mmc2.pdf]

Cell Reports
Medicine

Report

The combined HPV16-E2/E6/E7 T cell response in oropharyngeal cancer predicts superior survival

Graphical abstract

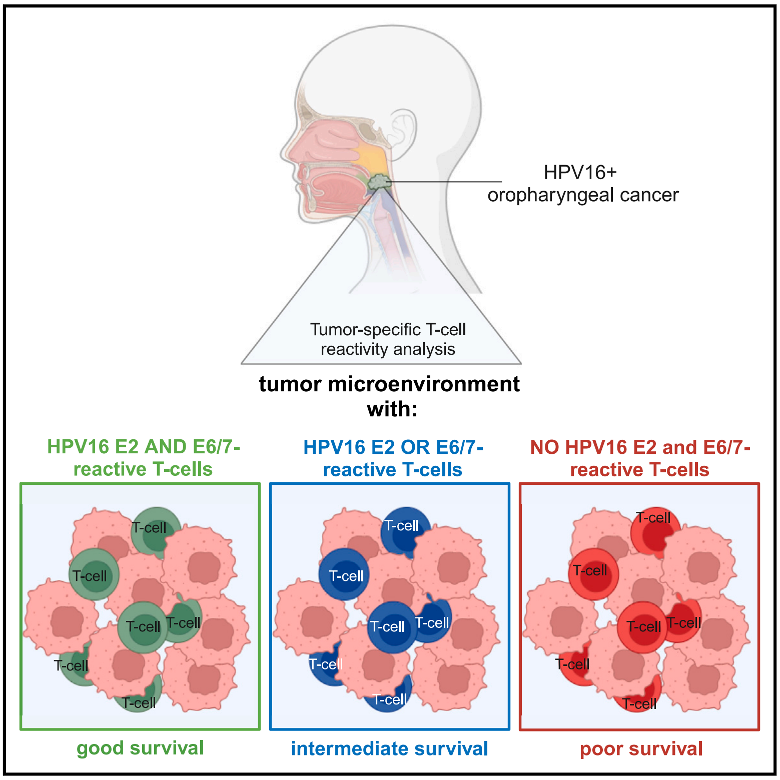

Highlights

- HPV16$^+$ OPSCC may contain T cells reactive to the viral proteins E6, E7, and E2
- These HPV16-reactive T cells display a polyfunctional type 1 cytokine profile
- Patients with intratumoral T cells to all 3 proteins display superior survival

Authors

Saskia J. Santegoets, Anouk Stolk, Marij J.P. Welters, Sjoerd H. van der Burg

Correspondence

shvdburg@lumc.nl

In brief

The infiltration of human papillomavirus (HPV)-induced oropharyngeal cancer by tumor-reactive T cells is important for treatment response. Santegoets et al. describe that in addition to E6 and E7, a third HPV protein, called E2, is targeted by tumor-infiltrating T cells and that these cells contribute to better clinical outcome.

Santegoets et al., 2023, Cell Reports Medicine *4*, 101262
November 21, 2023 © 2023 The Author(s).
https://doi.org/10.1016/j.xcrm.2023.101262

CellPress

Cell Reports Medicine

CellPress
OPEN ACCESS

Report

The combined HPV16-E2/E6/E7 T cell response in oropharyngeal cancer predicts superior survival

Saskia J. Santegoets,[1] Anouk Stolk,[1] Marij J.P. Welters,[1] and Sjoerd H. van der Burg[1,2,*]
[1]Department of Medical Oncology, Oncode Institute, Leiden University Medical Center, Albinusdreef 2, 2333 ZA Leiden, the Netherlands
[2]Lead contact
*Correspondence: shvdburg@lumc.nl
https://doi.org/10.1016/j.xcrm.2023.101262

SUMMARY

Tumor-infiltrating HPV16-E2-specific CD8^{+} T cells have been detected in HPV16-induced oropharyngeal squamous cell carcinoma (OPSCC). Whether intratumoral CD4^{+} T cells target HPV16 E2 and if HPV16-E2-specific immunity contributes to better clinical outcome is unknown. In a prospective HPV16^{+} OPSCC cohort, we regularly detect HPV16-E2-specific CD4^{+} and CD8^{+} intratumoral T cells, albeit at lower frequencies than the co-infiltrating HPV16-E6/E7-specific T cells. These HPV16-reactive T cells produce multiple cytokines when activated, indicating their polyfunctionality. Importantly, their combined intratumoral presence predicts superior survival, emphasizing the value of HPV16-E2-specific T cells in anti-tumor immunity and suggests its use as a target antigen for immunotherapy.

INTRODUCTION

Oropharyngeal squamous cell carcinoma (OPSCC) accounts for 0.5% of all cancer cases with ~98,000 new cases each year.[1] Between 50% and 70% of all OPSCCs are caused by human papillomavirus (HPV), predominantly after infection with HPV type 16 (HPV16).[2] Immune checkpoint inhibition (ICI) has been approved for treatment of recurrent/metastatic OPSCC but has met with limited clinical success.[3]

Early studies on HPV-driven carcinogenesis in cervical cancer (CxCa) revealed a multistep process of persistent HPV infection with progression from pre-cancerous to malignant lesions. In this process, the episomal DNA of HPV becomes integrated into the host genome,[4] leading to disruption of E2 gene expression and subsequent increased expression of the oncogenes E6 and E7. As a result, different immunotherapeutic strategies predominantly focused on targeting HPV E6 and E7.[3] Recent evidence shows that HPV-induced carcinogenesis of OPSCC and CxCa is not alike. In OPSCC, HPV16 is frequently found in a hybrid episomal form,[5–7] suggesting that other (early) HPV proteins may also form antigens in OPSCC. Indeed, multiple studies have shown E1, E2, E4, and E5 expression as well as high titers of serum antibodies and circulating T cell responses to E1, E2, E4, and E5 in OPSCC, supporting the persistent presentation and highlighting the potential immunogenicity of these early antigens in HPV16^{+} OPSCC.[8–12] This notion is in line with two recent studies showing the presence of tumor-infiltrating E2-specific CD8^{+} T cells in OPSCC.[13,14] Important questions arising from these studies are whether E2 is also a major target for intratumoral CD4^{+} T cells, whether E2-specific T cells contribute to a better clinical outcome, and how this response compares to T cell reactivity against the oncogenes E6 and E7. The latter is of particular interest since we recently demonstrated a strong role for CD4^{+} and CD8^{+} E6- and/or E7-reactive T cells[15] in tumor control and survival for patients with HPV16^{+} OPSCC tumors.

Here, we studied the presence and potential clinical impact of intratumoral HPV16 E2-specific CD4^{+} and CD8^{+} T cells in our previously described prospective HPV16^{+} OPSCC cohort with over 10 years of follow-up[15,16] and for which the intratumoral presence of HPV16-E6- and/or E7-specific T cells was already determined.

RESULTS AND DISCUSSION

HPV16 E2-specific CD4^{+} and CD8^{+} T cells are frequently found in tumor-infiltrating lymphocytes of HPV16^{+} OPSCC patients

HPV16-E2-reactive CD4^{+} T cells could be detected in 21 of 35 IL-2 expanded tumor-infiltrating lymphocyte (TIL) cultures of HPV16^{+} OPSCC patients. In 19 cases, E2-reactivity coincided with E6/E7-reactive CD4^{+} T cells, in 2 cases, only E2-reactivity was found, and in 6 cases, only a response to E6/E7 was found (Figures 1A and 1B). E2-reactive CD4^{+} T cells were detected at lower levels than E6/E7-reactive CD4^{+} T cells (Figure 1A). Notably, E2-reactive CD8^{+} T cells were found in about half of the cases showing CD4^{+} T cell reactivity, especially when tumors were infiltrated by both E2- and E6/E7-specific CD4^{+} T cells (Figures 1C–1E). The reactivity of the CD4^{+} and CD8^{+} T cells was against epitopes throughout the entire HPV16 E2 protein and not dominated by one region (Figures 1F–1I). As dictated by the break in the E2 gene in CxCa,[6] HPV16-E2 reactivity was less often detected in HPV16^{+} CxCa samples with evident E6/E7 reactivity (Figures S1A and S1D), focused only on its N-terminal part (Figures S1B and S1E), and present at much lower levels than in OPSCC tumors, while this was not the case for E6/E7-specific T cells (Figures S1C and S1F).

This is an open access article under the CC BY license (http://creativecommons.org/licenses/by/4.0/).

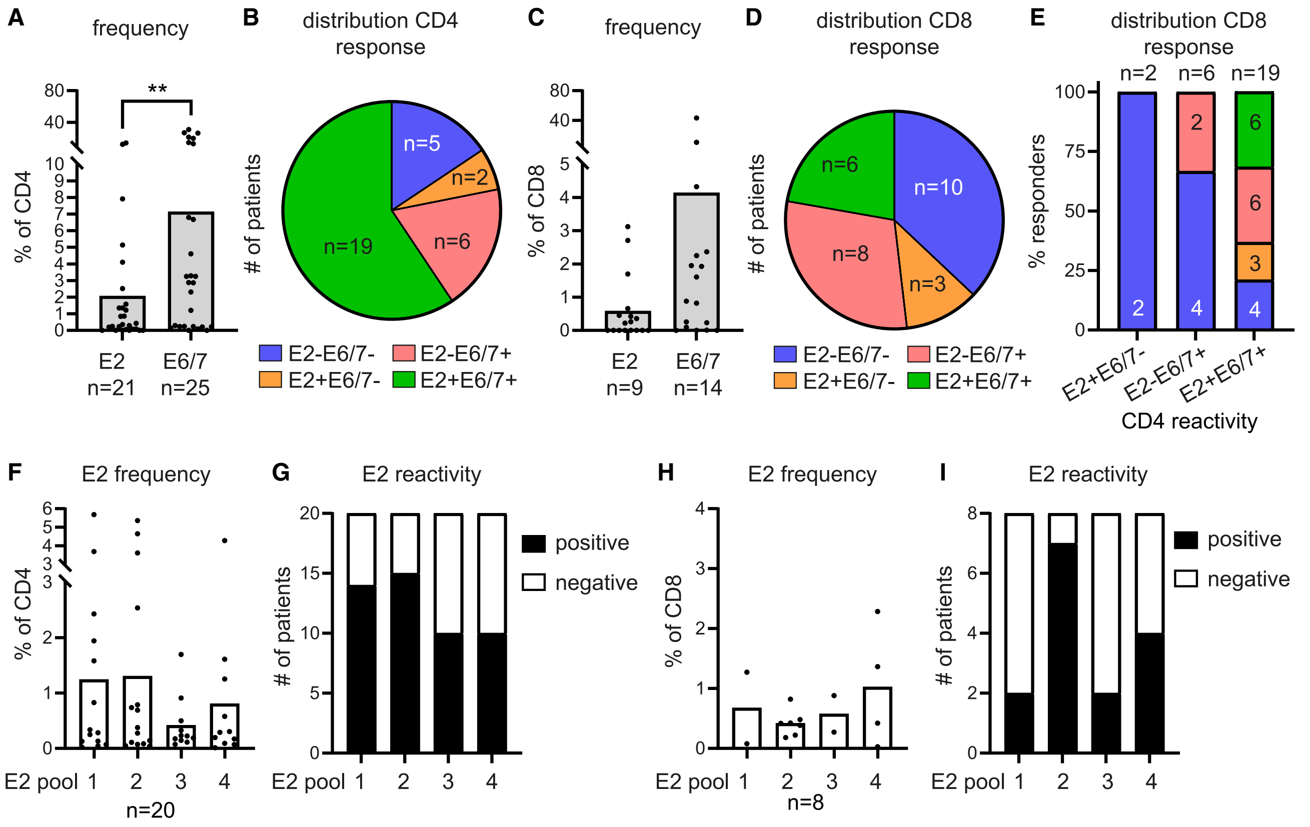

Figure 1. HPV16 E2-specific CD4$^+$ and CD8$^+$ T cells can be found in TILs of OPSCC patients

Intracellular cytokine-staining analysis by flow cytometry with antibodies against CD3, CD4, CD8, CD137, CD154, TNFα, GM-CSF, IFNγ, IL-2, and CCL4.

(A and C) Percentage of total E2- and E6/E7-reactive CD4$^+$ (A) and CD8$^+$ (C) T cells.

(B and D) Number of patients with CD4$^+$ (B) and CD8$^+$ (D) T cell reactivity to either E2 and/or E6/7.

(E) Percentage of patients with CD8$^+$ T cell reactivity to either E2 and/or E6/E7 coinciding with E2+E6/E7−, E2−E6/E7+, or E2+E6/E7+ CD4$^+$ T cell reactivity (horizontal axis, left to right). Number of patients is given in the bar graph.

(F–I) Percentage of E2-reactive CD4$^+$ (F) and CD8$^+$ T cells (H) and the distribution of detectable E2-reactive CD4$^+$ T cells (G) and CD8$^+$ T cells (I) among patients for the E2 peptide-containing pools 1 to 4. See also Figure S1.

Two-tailed paired t tests were used for statistical analysis. *$p < 0.05$, **$p < 0.01$, ***$p < 0.001$, and ****$p < 0.0001$.

HPV16 E2-, E6-, and E7-specific CD8$^+$ and CD4$^+$ type 1 T cells in OPSCC are polyfunctional

To gain insight into the quality of the response, determined by the polyfunctionality of the E2-and E6/7-reactive T cells, in-depth single-cell high-dimensional clustering and trajectory interference analysis was performed. HPV16-E2- and HPV16-E6/E7-reactive CD4$^+$ and CD8$^+$ T cells were selected on antigen-specific expression of the activation markers CD137 and/or CD154 (Figures S1G–S1I), yielding 245,072 activated CD4$^+$ T cells and 47,317 activated CD8$^+$ T cells. Dimensionality reduction using Uniform Manifold Approximation and Projection (UMAP) analysis followed by FlowSOM consensus metaclustering revealed that HPV16-reactive T cells are capable of producing multiple cytokines up to a total of 5 simultaneously (Figures 2A, S2A, S2B, and S2E–S2G). To quantify polyfunctionality, the HPV16-reactive CD4$^+$ and CD8$^+$ T cells were subsequently subjected to manual gating, identifying 32 different cytokine populations based on the expression of typical type 1 cytokines TNFα, GM-CSF, IFNγ, IL-2, and CCL4 and combinations thereof (Figures S1G–S1I; Table S1). HPV16 E2-, E6-, E7-specific T cells in OPSCC and CxCa are polyfunctional, albeit that more polyfunctional T cells were detected among the E6/E7-reactive population (Figures 2B, S2H, and S2I). The trajectory algorithm Wanderlust predicted that the developmental path for CD4$^+$ T cells progresses from early activation (i.e., CD154 expression) with fast production of TNFα and GM-CSF to full-blown activation with additional production of IFNγ, IL-2, and CCL4 (Figure S2C), whereas CD8$^+$ T cells progress from early activation (i.e., CD137 expression) with fast production of TNFα to full-blown activation with CD154 expression and production of GM-CSF, IFNγ, IL-2, and CCL4 (Figure S2D).

The presence of E2-, E6-, and E7-specific CD8$^+$ and CD4$^+$ T cells is associated with superior survival in OPSCC

Finally, to determine the clinical impact of intratumoral E2-reactive T cells and compare it to the impact of E6/E7-reactive T cells, survival analyses were performed (Figures 3 and S3). Clearly,

CellPress
OPEN ACCESS

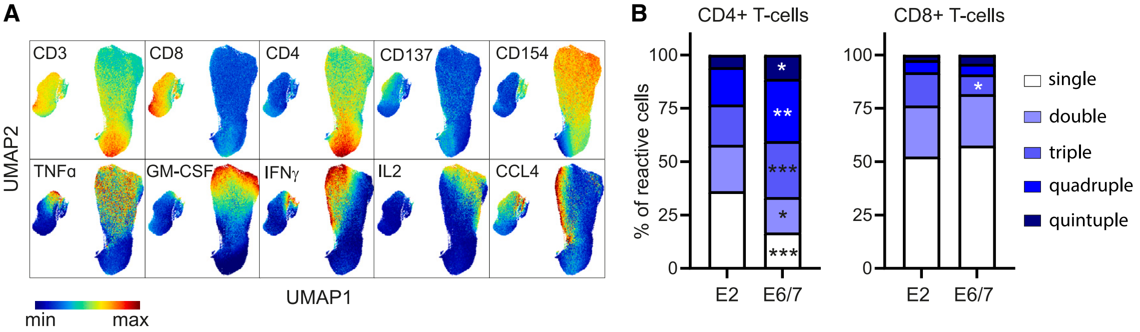

Figure 2. HPV16 E2-, E6-, and E7-specific CD8+ and CD4+ T cells in OPSCC are polyfunctional

High-dimensional single-cell data analysis of 116 activated CD4+ and activated CD8+ T cell populations was performed using OMIQ software.

(A) Expression intensity of the cell surface and cytokine markers on the UMAP plot.

(B) Percentage of single, double, triple, quadruple, and quintuple cytokine-producing CD4+ T cells (left) and CD8+ T cells (right) for the total E2- and E6/E7-reactive T cell populations. See also Figures S1 and S2 and Table S1.

Two-tailed paired t tests were used for statistical analysis. *$p < 0.05$, **$p < 0.01$, ***$p < 0.001$, and ****$p < 0.0001$.

while the presence of intratumoral E6- and/or E7-reactive T cells alone already excellently predicts improved survival for patients with HPV16+ OPSCC (Figure 3A),[15] the presence of HPV16-E2-specific T cell reactivity further improves survival, as HPV16+ OPSCC patients with T cells specifically producing type 1 cytokines to both E2 and E6/E7 display superior survival (Figures 3B, S3B, and S3C). Interestingly, there was a trend toward increased survival for patients with a relatively higher percentage (>median) of HPV16 E2/E6/E7-specific cytokine-producing T cells among the IR-positive patients (Figure S3D). A clear difference in survival was also observed when the presence of HPV16 E2/E6/E7-specific T cells was scored solely on basis of antigen-specific induced expression of the activation molecules CD137 and CD154 (Figure S3E), albeit that some of the responses detected by cytokine production were lost (Figure S3G) due to a higher background expression of CD137 and/or CD154 on non-stimulated T cells, leading to 5 IR false-negative scored patients. The capacity of TILs to respond to the general T cell activator phytohemagglutinin was not distinctive in predicting survival (Figure S3F).

In view of the well-known role of type-1 CD4+ T cells in optimal CTL priming, T memory cell expansion, tumor immune microenvironment optimization, and stimulation of intratumoral innate effector cells,[17] and in combination with the demonstration that E2-specific CD8+ can kill HPV16+ OPSCC cells,[18] our study emphasizes the value of targeting HPV16-E2 in addition to HPV16-E6 and -E7 when developing immunotherapeutic strategies for HPV16+ OPSCC to improve the current efficacy of ICI therapy in these cancers. Potential benefit of combining HPV16 E6/E7 vaccination with ICI has been recently demonstrated by Massarelli et al.,[19,20] showing signs of prolonged overall response rates and overall survival compared with ICI therapy alone in similar patients. Our observation that E2-specific T cells may impact survival suggests that incorporation of E2 as a target antigen into this or other vaccines could increase the proportion of HPV16+ OPSCC patients benefiting from ICI therapy.

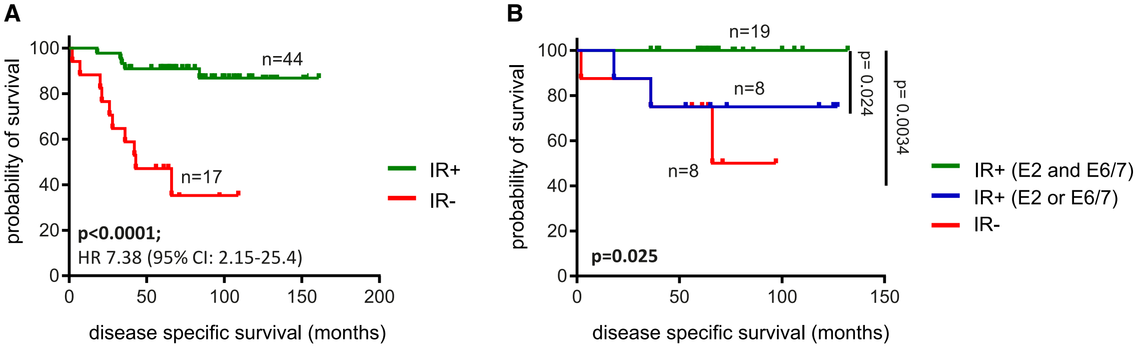

Figure 3. The presence of E2-, E6-, and E7-specific CD8+ and CD4+ T cells is associated with superior survival in OPSCC

Kaplan-Meier survival curves of HPV16+ OPSCC patients (A) with (IR+; green) or without (IR–; red) an intratumoral HPV16-E2- and/or E6/7-specific T cell response as determined by proliferation and cytokine assay and intracellular cytokine-staining (ICS) analysis and (B) with both intratumoral HPV16-E2- and E6/E7-specific T cells (IR+; green) versus HPV16 E2- or E6/E7-specific T cells only (IR+; blue) versus no HPV16-specific T cells (IR–; red) as determined by ICS. See also Figure S3.

Non-parametric log-rank Mantel-Coxt tests were used for statistical analysis.

LIMITATIONS OF THE STUDY

Although we report that the combined presence of E2-, E6-, and E7-specific CD8$^+$ and CD4$^+$ T cells is associated with superior survival in OPSCC and emphasize the value of HPV16-E2-specific T cells in anti-tumor immunity, we have not assessed the actual expression of these HPV antigens in the tumors of patients in which these responses were detected or tested the direct recognition of tumor cell by the TIL. Furthermore, our analysis was restricted to the measurement of responses to the three early proteins E2, E6, and E7 of HPV, neglecting the possibility that TIL may also respond to the other early proteins E1, E4, and/or E5 to which circulating T cells have been detected in OPSCC.[12] Consequently, some patients defined as immune response negative may have been mislabeled. Finally, E2, E6, and E7 reactivity and polyfunctionality was not determined in directly *ex vivo* isolated TIL, but only after *in vitro* expansion with IL-2. We can't exclude that the *in vitro* expansion of specific TIL differs among patients due to differences in the intrinsic *in vitro* expansion capacity of T cell clones present among the TIL. The conclusions about the relative difference in magnitude of the E2, E6, and E7 response versus survival should, therefore, be interpreted with caution.

STAR★METHODS

Detailed methods are provided in the online version of this paper and include the following:

- KEY RESOURCES TABLE
- RESOURCE AVAILABILITY
  - Lead contact
  - Materials availability
  - Data and code availability
- EXPERIMENTAL MODEL AND SUBJECT DETAIL
  - Oropharyngeal cancer and cervical cancer patient cohorts
- METHOD DETAILS
  - Blood and tumor cell isolation and TIL culturing
  - Tumor-specific T cell reactivity analysis by intracellular cytokine staining (ICS)
- QUANTIFICATION AND STATISTICAL ANALYSIS
  - Statistical analysis

SUPPLEMENTAL INFORMATION

Supplemental information can be found online at https://doi.org/10.1016/j.xcrm.2023.101262.

ACKNOWLEDGMENTS

S.H.v.d.B. is financed via the Oncode Base Fund of the Oncode Institute.

AUTHOR CONTRIBUTIONS

Conceptualization: S.J.S., M.J.P.W., and S.H.v.d.B.; data acquisition: S.J.S. and A.S.; data analysis: S.J.S.; writing – original draft: S.J.S. and S.H.v.d.B.; writing – review and editing S.J.S., M.J.P.W., and S.H.v.d.B.

DECLARATION OF INTERESTS

S.H.v.d.B. is a paid member of the Strategic Advisory Board of ISA pharmaceuticals, a company developing a therapeutic vaccine against HPV16. S.H.v.d.B. is mentioned as inventor on several patents concerning therapeutic HPV-specific vaccines and how to apply them.

Received: May 3, 2023
Revised: August 2, 2023
Accepted: October 6, 2023
Published: November 3, 2023

REFERENCES

1. Sung, H., Ferlay, J., Siegel, R.L., Laversanne, M., Soerjomataram, I., Jemal, A., and Bray, F. (2021). Global Cancer Statistics 2020: GLOBOCAN Estimates of Incidence and Mortality Worldwide for 36 Cancers in 185 Countries. CA. Cancer J. Clin. *71*, 209–249. https://doi.org/10.3322/caac.21660.
2. Gillison, M.L., Chaturvedi, A.K., Anderson, W.F., and Fakhry, C. (2015). Epidemiology of Human Papillomavirus-Positive Head and Neck Squamous Cell Carcinoma. J. Clin. Oncol. *33*, 3235–3242. https://doi.org/10.1200/jco.2015.61.6995.
3. Roof, L., and Yilmaz, E. (2023). Immunotherapy in HPV-Related Oropharyngeal Cancers. Curr. Treat. Options Oncol. *24*, 170–183. https://doi.org/10.1007/s11864-023-01050-x.
4. Cullen, A.P., Reid, R., Campion, M., and Lörincz, A.T. (1991). Analysis of the physical state of different human papillomavirus DNAs in intraepithelial and invasive cervical neoplasm. J. Virol. *65*, 606–612. https://doi.org/10.1128/jvi.65.2.606-612.1991.
5. Nulton, T.J., Olex, A.L., Dozmorov, M., Morgan, I.M., and Windle, B. (2017). Analysis of The Cancer Genome Atlas sequencing data reveals novel properties of the human papillomavirus 16 genome in head and neck squamous cell carcinoma. Oncotarget *8*, 17684–17699. https://doi.org/10.18632/oncotarget.15179.
6. Schrank, T.P., Kim, S., Rehmani, H., Kothari, A., Wu, D., Yarbrough, W.G., and Issaeva, N. (2022). Direct Comparison of HPV16 Viral Genomic Integration, Copy Loss, and Structural Variants in Oropharyngeal and Uterine Cervical Cancers Reveal Distinct Relationships to E2 Disruption and Somatic Alteration. Cancers *14*, 4488. https://doi.org/10.3390/cancers14184488.
7. Morgan, I.M., DiNardo, L.J., and Windle, B. (2017). Integration of Human Papillomavirus Genomes in Head and Neck Cancer: Is It Time to Consider a Paradigm Shift? Viruses *9*, 208. https://doi.org/10.3390/v9080208.
8. Anderson, K.S., Gerber, J.E., D'Souza, G., Pai, S.I., Cheng, J.N., Alam, R., Kesiraju, S., Chowell, D., Gross, N.D., Haddad, R., et al. (2015). Biologic predictors of serologic responses to HPV in oropharyngeal cancer: The HOTSPOT study. Oral Oncol. *51*, 751–758. https://doi.org/10.1016/j.oraloncology.2015.05.007.
9. Ramqvist, T., Mints, M., Tertipis, N., Näsman, A., Romanitan, M., and Dalianis, T. (2015). Studies on human papillomavirus (HPV) 16 E2, E5 and E7 mRNA in HPV-positive tonsillar and base of tongue cancer in relation to clinical outcome and immunological parameters. Oral Oncol. *51*, 1126–1131. https://doi.org/10.1016/j.oraloncology.2015.09.007.
10. Zhang, Y., Koneva, L.A., Virani, S., Arthur, A.E., Virani, A., Hall, P.B., Warden, C.D., Carey, T.E., Chepeha, D.B., Prince, M.E., et al. (2016). Subtypes of HPV-Positive Head and Neck Cancers Are Associated with HPV Characteristics, Copy Number Alterations, PIK3CA Mutation, and Pathway Signatures. Clin. Cancer Res. *22*, 4735–4745. https://doi.org/10.1158/1078-0432.Ccr-16-0323.
11. Kürten, C.H.L., Kulkarni, A., Cillo, A.R., Santos, P.M., Roble, A.K., Onkar, S., Reeder, C., Lang, S., Chen, X., Duvvuri, U., et al. (2021). Investigating immune and non-immune cell interactions in head and neck tumors by single-cell RNA sequencing. Nat. Commun. *12*, 7338. https://doi.org/10.1038/s41467-021-27619-4.

CellPress
OPEN ACCESS

12. Bhatt, K.H., Neller, M.A., Srihari, S., Crooks, P., Lekieffre, L., Aftab, B.T., Liu, H., Smith, C., Kenny, L., Porceddu, S., and Khanna, R. (2020). Profiling HPV-16-specific T cell responses reveals broad antigen reactivities in oropharyngeal cancer patients. J. Exp. Med. *217*, e20200389. https://doi.org/10.1084/jem.20200389.

13. Eberhardt, C.S., Kissick, H.T., Patel, M.R., Cardenas, M.A., Prokhnevska, N., Obeng, R.C., Nasti, T.H., Griffith, C.C., Im, S.J., Wang, X., et al. (2021). Functional HPV-specific PD-1(+) stem-like CD8 T cells in head and neck cancer. Nature *597*, 279–284. https://doi.org/10.1038/s41586-021-03862-z.

14. McInnis, C., Bhatia, S., Vijaykumar, B., Tian, Q., Sun, Y., Leistritz-Edwards, D., Quinn, C.T., Uppaluri, R., Egloff, A.M., Srinivasan, L., et al. (2023). Identification of HPV16 E1 and E2-specific T cells in the oropharyngeal cancer tumor microenvironment. J. Immunother. Cancer *11*, e006721. https://doi.org/10.1136/jitc-2023-006721.

15. Welters, M.J.P., Ma, W., Santegoets, S.J.A.M., Goedemans, R., Ehsan, I., Jordanova, E.S., van Ham, V.J., van Unen, V., Koning, F., van Egmond, S.I., et al. (2018). Intratumoral HPV16-Specific T Cells Constitute a Type I-Oriented Tumor Microenvironment to Improve Survival in HPV16-Driven Oropharyngeal Cancer. Clin. Cancer Res. *24*, 634–647. https://doi.org/10.1158/1078-0432.Ccr-17-2140.

16. Abdulrahman, Z., Santegoets, S.J., Sturm, G., Charoentong, P., Ijsselsteijn, M.E., Somarakis, A., Höllt, T., Finotello, F., Trajanoski, Z., van Egmond, S.L., et al. (2022). Tumor-specific T cells support chemokine-driven spatial organization of intratumoral immune microaggregates needed for long survival. J. Immunother. Cancer *10*, e004346. https://doi.org/10.1136/jitc-2021-004346.

17. Borst, J., Ahrends, T., Bąbała, N., Melief, C.J.M., and Kastenmüller, W. (2018). CD4(+) T cell help in cancer immunology and immunotherapy. Nat. Rev. Immunol. *18*, 635–647. https://doi.org/10.1038/s41577-018-0044-0.

18. Krishna, S., Ulrich, P., Wilson, E., Parikh, F., Narang, P., Yang, S., Read, A.K., Kim-Schulze, S., Park, J.G., Posner, M., et al. (2018). Human Papilloma Virus Specific Immunogenicity and Dysfunction of CD8(+) T Cells in Head and Neck Cancer. Cancer Res. *78*, 6159–6170. https://doi.org/10.1158/0008-5472.Can-18-0163.

19. Massarelli, E., William, W., Johnson, F., Kies, M., Ferrarotto, R., Guo, M., Feng, L., Lee, J.J., Tran, H., Kim, Y.U., et al. (2019). Combining Immune Checkpoint Blockade and Tumor-Specific Vaccine for Patients With Incurable Human Papillomavirus 16-Related Cancer: A Phase 2 Clinical Trial. JAMA Oncol. *5*, 67–73. https://doi.org/10.1001/jamaoncol.2018.4051.

20. (2021). FDA Gives ISA101b Fast Track Designation for HPV 16+ Oropharyngeal Cancer. News release. ISA Pharmaceuticals. September 14, Accessed July 18, 2023. Available online: https://www.cancernetwork.com/view/fda-gives-isa101b-fast-track-designation-for-hpv-16-oropharyngeal-cancer

21. Santegoets, S.J., van Ham, V.J., Ehsan, I., Charoentong, P., Duurland, C.L., van Unen, V., Höllt, T., van der Velden, L.A., van Egmond, S.L., Kortekaas, K.E., et al. (2019). The Anatomical Location Shapes the Immune Infiltrate in Tumors of Same Etiology and Affects Survival. Clin. Cancer Res. *25*, 240–252. https://doi.org/10.1158/1078-0432.Ccr-18-1749.

22. de Vos van Steenwijk, P.J., Heusinkveld, M., Ramwadhdoebe, T.H., Löwik, M.J., van der Hulst, J.M., Goedemans, R., Piersma, S.J., Kenter, G.G., and van der Burg, S.H. (2010). An unexpectedly large polyclonal repertoire of HPV-specific T cells is poised for action in patients with cervical cancer. Cancer Res. *70*, 2707–2717. https://doi.org/10.1158/0008-5472.Can-09-4299.

STAR★METHODS

KEY RESOURCES TABLE

REAGENT or RESOURCE	SOURCE	IDENTIFIER
Antibodies		
Anti-human CD8 Brilliant Blue700 (clone HIT8a)	BD biosciences	cat# 742229; RRID: AB_2740667
Anti-human GM-CSF APC (clone BVD2-21C11	Biolegend	cat# 502310; RRID: AB_11150231
Anti-human IFNγ Alexa Fluor 700 (clone B27)	BD biosciences	cat# 557995; RRID: AB_396977
Anti-human CD137 Brilliant Violet421 (clone 4B4-1)	BD biosciences	cat# 564091; RRID: AB_2722503
Anti-human CD3 Brilliant Violet 510 (clone UCHT1)	Biolegend	cat# 300448; RRID: AB_2563468
Anti-human TNFα Brilliant Violet 605 (clone Mab11)	Biolegend	cat# 502936; RRID: AB_2563884
Anti-human CD4 Brilliant Violet 650 (clone RPA-T4)	Biolegend	cat# 300536; RRID: AB_2632791
Anti-human MIP-1 beta (CCL4) PE (clone D21-1351)	BD biosciences	cat# 550078; RRID: AB_393549
Anti-human CD154 PE-CF594 (clone TRAP1)	BD biosciences	cat# 563589; RRID: AB_2738297
Anti-human IL-2 PE-Cy7 (clone MQ1-17H1)	Biolegend	cat# 500326; RRID: AB_2125593
Biological samples		
Oropharyngeal Squamous Cell Carcinoma (OPSCC)	Leiden University Medical Center	N/A
Cervical Carcinoma (CxCa)	Leiden University Medical Center	N/A
Chemicals, peptides, and recombinant proteins		
Brefeldin A	Sigma-Aldrich	B7651
Interleukin-2 (Aldesleukin)	Novartis	RVG 13354
Paraformaldehyde	Sigma-Aldrich	P6148
HPV16 E2, E6 and E7 peptides	Peptide synthesis facility of department IHB, LUMC	N/A
Saponin	Sigma-Aldrich	S7900
Critical commercial assays		
LIVE/DEAD™ Fixable Near-IR dead cell stain kit	ThermoFisher	L10119
Software and algorithms		
FlowJo v10	Tree Star, Inc.	www.flowjo.com
OMIQ	Omiq Inc (CA, USA)	www.omiq.ai
GraphPad Prism v9	GraphPad Software	https://www.graphpad.com

RESOURCE AVAILABILITY

Lead contact

Further information and requests for resources and reagents should be directed to the lead contact, Professor Dr. Sjoerd H. van der Burg (shvdburg@lumc.nl).

Materials availability

This study did not generate new unique reagents.

Data and code availability

- All data supporting the findings of this study are available from the lead contact upon reasonable request.
- This report does not report original code.
- Any additional information required to reanalyze the data reported in this paper is available from the lead contact upon reasonable request

EXPERIMENTAL MODEL AND SUBJECT DETAIL

Oropharyngeal cancer and cervical cancer patient cohorts

Patients included in this study were part of 2 larger prospective observational studies om oropharyngeal squamous cell carcinoma (OPSCC) and cervical carcinoma (CxCa). Patients with histologically confirmed OPSCC were included in a study investigating the circulating and local immune response in patients with head and neck cancer (P07-112) and women with histologically proven cervical carcinoma were included in the CIRCLE study investigating cellular immunity against anogenital lesions. Patients were included after signing an informed consent.[15,16,21,22] Both studies were conducted in accordance with the Declaration of Helsinki and approved by the local medical ethical committee of the Leiden University Medical Center (LUMC) and in agreement with the Dutch law. All tumor material and blood were sampled prior to therapy. Patients were treated with standard of care therapy including surgery, radiotherapy, chemotherapy or a combination thereof. Details on age, gender, tumor location and treatment received are provided in Table S2 p16INK4a staining and HPV typing was done on formalin-fixed paraffin-embedded tumor tissue according to standard diagnostic procedures at the LUMC department of Pathology as described.[15,21] Survival of the patients was updated until March 2023.

In this study, a total of 61 TIL batches from OPSCC patients were analyzed. This number was based on availability of expanded TIL samples of HPV16+ tumors and autologous monocytes and/or EBV-transformed B-lymphoblastoid cells (EBV-B).

METHOD DETAILS

Blood and tumor cell isolation and TIL culturing

Blood samples were drawn in sodium-heparin collection tubes (Vacuette; Greiner, Alphen a/d Rijn, the Netherlands) prior to surgery. PBMC were isolated by ficoll density centrifugation, washed in PBS, frozen in 90% FCS (PAA laboratories) supplemented with 10% DMSO (Sigma) and stored in liquid nitrogen until use. Tumor biopsies were obtained from the operating theater and handled as described previously.[15,16,21] In brief, tumor material was cut into small pieces, and subsequently incubated for 60 min at 37°C in IMDM (Lonza) with 10% human AB serum (Capricorn Scientific) and supplemented with high dose antibiotics (50 μg/mL Gentamycin, 25 μg/mL Fungizone, 100 IU/mL penicillin and 100 μg/mL streptomycin (all ThermoFisher Scientific, Bleiswijk, the Netherlands). Subsequently, TIL were cultured from the tumor pieces by culturing them in IMDM supplemented with 10% human AB serum, 100 IU/mL penicillin, 100 μg/mL streptomycin, 2 mmol/L L-glutamin (Lonza; IMDM complete), and 1,000 IU/mL human recombinant IL2 (Aldesleukin, Novartis). Cultures were replenished every 2 to 3 days with fresh IMDM complete and IL2 to a final concentration of 1000 IU/mL. When there were sufficient T cells, the cells were cryopreserved and stored in liquid nitrogen until use.

Tumor-specific T cell reactivity analysis by intracellular cytokine staining (ICS)

To determine the specificity of the tumor infiltrating T cells in HPV16+ OPSCC, cultured TIL batches were analyzed for the presence HPV16 E6 and/or E7-reactive T cells using a combined [3H]-thymidine-based proliferation and cytokine production assay as described previously.[15,16,21] To this end, monocytes were isolated from autologous PBMC through plastic adherence for 2 h, after which non-adherent cells were discarded and the adherent monocytes were cultured for 48–72 h with X-vivo 15 medium (Lonza) supplemented with 800 U/mL of GM-CSF. Next, monocytes were loaded overnight with 5 μg/mL HPV16 E6/E7 synthetic long peptides, after which the excess peptide was removed, and the cells were used to stimulated the TIL in triplicate for 5 days 0.5 μg/mL PHA (HA16 Remel, Thermofisher) and non-loaded (medium) monocytes served as positive and negative control respectively. At day 1.5 and 4, supernatant was harvested to determine cytokine production, which was done by cytometric bead array (Th1/Th2 kit, BD Biosciences) according to manufacturer's instructions. During the last 16 h, 0.5 μCi/well of [3H]-thymidine was added to measure proliferation. A response was considered positive when the stimulation index (average of test wells divided by the average of the medium control wells) of proliferation was at least 3 and/or cytokine production was at least twice above the medium control and above the assays cut-off value. Assessment of HPV16 E2-reactivity was tested by intracellular cytokine staining (ICS) by flow cytometry on a subset of these TIL batches, and selection was based on availability of TIL batches and PBMC or EBV-transformed B-lymphoblastoid cell (EBV-B). To this end, reactivity of the cultured TILs was tested using autologous monocytes (prepared as described above) or EBV-B cells loaded with 5 μg/mL HPV16 E2 SLPs as well as HPV16 E6/E7 SLPs in parallel, thereby allowing for a direct comparison in reactivity between E2 and E6/E7. Non-loaded monocytes or EBV-B cells served as negative control to establish background reactivity of the T cells. The HPV16 E2 peptides were 30-mer peptides with 15 amino acids overlap that were divided into 4 different pools: *E2-p1*: 1–30, 16–45, 31–60, 46–75, 61–90, 76–105; *E2-p2*: 91–120, 106–135,121–150, 136–165, 151–180, 166–195; *E2-p3*: 181–210, 196–225, 211–240, 226–255, 241–270, 256–285; *E2-p4:* 271–300, 286–315, 286–315, 301–330, 316–345, 331–365. The HPV16 E6 and E7 peptides consisted of 22-mer peptides with 14 amino acids overlap that were put together into 1 pool: *E6:* 1–22, 11–32, 21–42, 31–52, 41–62, 51–72, 61–82, 71–92, 81–102, 91–112, 101–122, 111–132, 111–132, 121–142, 131–152, 137–158 and *E7:* 1–22, 11–32, 21–42, 31–52, 41–62, 51–72, 61–82, 71–92, 77–98. ICS was performed with antibodies directed against CD3 (UCHT1), CD4 (RPA-T4), TNFα (MAb11), GM-CSF (BVD2-21C11), IL-2 (MQ1-17H1; all from Biolegend), CD8 (HIT8a), CD137 (4B4-1), CD154 (TRAP1), IFNγ (B27) and CCL4 (D21-1351; all from BD Biosciences) following overnight stimulation with SLP-loaded autologous monocytes or EBV-B cells in the presence of 10 μg/mL Brefeldin A (Sigma-Aldrich). Acquisition was done on an LSRII fortessa (BD biosciences). Specific cytokine-producing T cells were identified by manual gating using FlowJo software V10.8.1. Cells

were gated for live, single cells, CD3, CD4 and CD8 expression. Activated $CD4^+$ and $CD8^+$ cells were selected based on CD137 and/or CD154 expression and further analyzed for TNFα, GM-CSF, IFNγ, IL2 and CCL4 expression. To this end, cells were first gated for TNFα and GM-CSF, yielding TNFα+GM-CSF-, TNFα+GM-CSF+, TNFα-GM-CSF+ and TNFα-GM-CSF- populations. Next, these four populations were gated for IFNγ and IL2 (i.e., IFNγ+ IL2-, IFNγ+IL2+, IFNγ-IL2+ and IFNγ-IL2-populations), followed by subsequent gating on CD4 or CD8 and CCL4. Cytokine gates were set on total $CD4^+$ and $CD8^+$ T cells (reference plots). A gating example for this sequential gating is depicted in Figures S1G–S1I. All possible cytokine combinations are given in Table S1. A positive response was defined as at least two times the value of the negative control, and at least 10 positive spots in the gate for any of the 5 cytokines analyzed. The total frequency of E2 and E6/E7-reactive T cells is calculated as the SUM of all possible cytokine combinations as depicted in Table S1. Cytokine-producing T cells were also analyzed by high-dimensional single cell data analysis via Unifold Manifold Approximation and Projection (UMAP) dimensionality reduction followed by FlowSOM consensus metaclustering and wanderlust trajectory analysis using the cloud-based OMIQ data analysis software. To this end, HPV16 E2, E6 and/or E7-reactive T cells were selected from TIL cultures by manual gating on CD137 and/or CD154 ($CD4^-$and CD8-activated T cell populations as above) following stimulation with E2, E6/E7-loaded autologous monocytes or EBV B cells. Next, the newly formed FCS files containing these HPV16 E2, E6 and/or E7-reactive T cells were used for analysis by UMAP, FlowSOM and wanderlust trajectory analysis using OMIQ. The obtained different cell populations were visualized and quantified in OMIQ and after exporting the counts, graphs were generated using Graphpad prism V9.5.1 (Graphpad software, LA Jolla, California, USA).

QUANTIFICATION AND STATISTICAL ANALYSIS

Statistical analysis

Statistical was performed using. Differences between frequencies of E2 and E6/E7-reactive T cells were compared using a two-tailed paired T-test. Differences in frequencies of reactive T cells between OPSCC and CxCa patients were compared using the Mann-Whitney test. Differences in survival were calculated with the non-parametric log rank Mantel-Cox test. Grouping was done based on the detection of an intratumoral HPV16-specific T cell response (i.e., immune response (IR)-positive) or no detectable HPV16-specific immune response (i.e., IR-negative). A patient was scored as IR-positive when E2, E6 and/or E7-specific T cells were detected by antigen-specific proliferation and/or cytokine production (cytokine bead array/ICS). Differences were considered significant when $p < 0.05$. Statistical details of experiments such as n value, information on grouping for survival analysis and level of statistical significance (*, $p < 0.05$; **, $p < 0.01$; ***, $p < 0.001$; and ****, $p < 0.0001$) can be found in the figures and/or figure legends.

Cell Reports Medicine, Volume *4*

Supplemental information

The combined HPV16-E2/E6/E7 T cell response

in oropharyngeal cancer predicts superior survival

Saskia J. Santegoets, Anouk Stolk, Marij J.P. Welters, and Sjoerd H. van der Burg

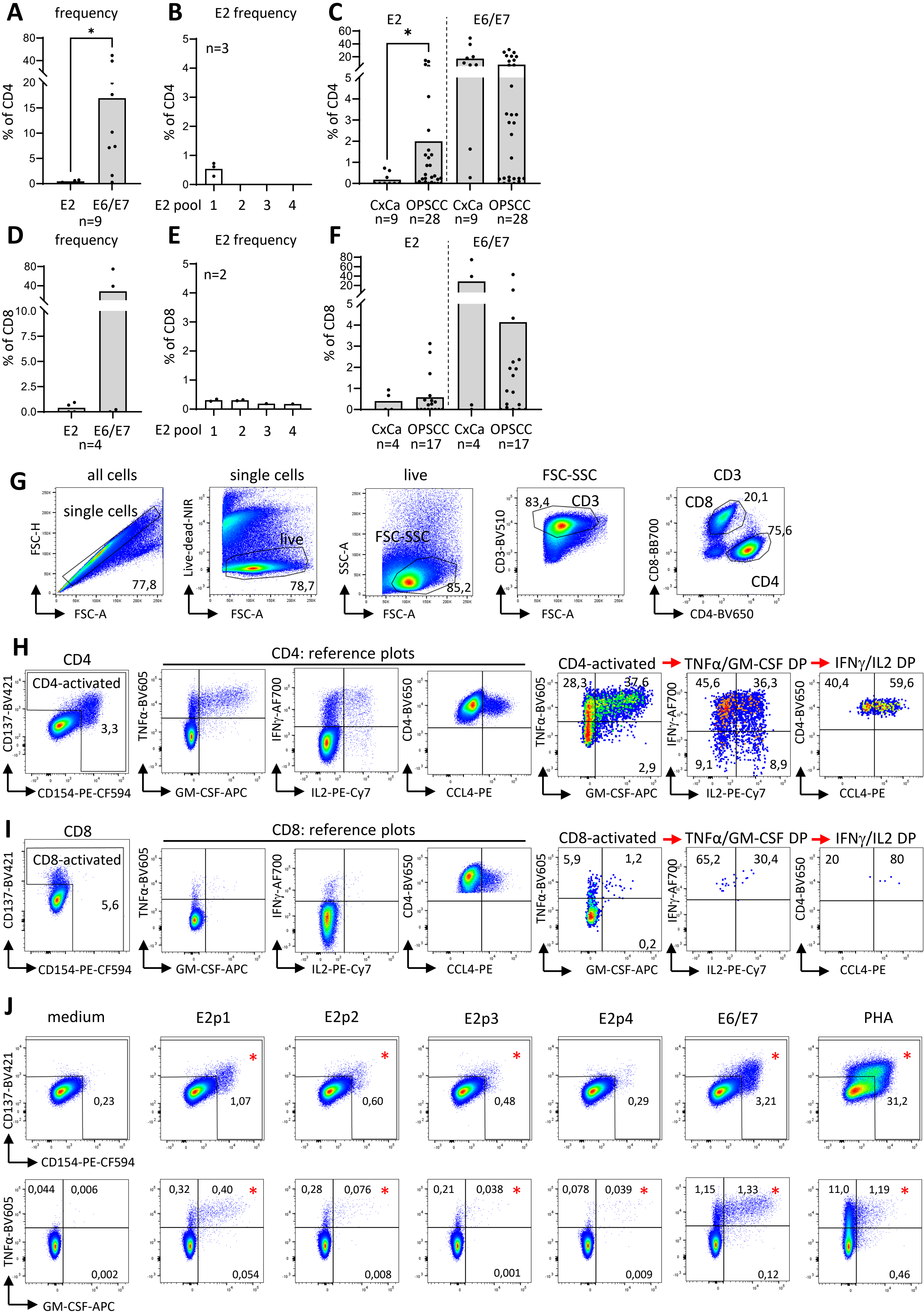
A
frequency
% of CD4
E2
E6/E7
n=9
B
E2 frequency
n=3
E2 pool 1 2 3 4
C
E2
E6/E7
CxCa n=9
OPSCC n=28
D
frequency
% of CD8
n=4
E
E2 frequency
n=2
F
CxCa n=4
OPSCC n=17
G
all cells
single cells
live
FSC-SSC
CD3
77,8
78,7
85,2
83,4
20,1
75,6
CD8
CD4
FSC-H
FSC-A
Live-dead-NIR
SSC-A
CD3-BV510
CD8-BB700
CD4-BV650
H
CD4
CD4-activated
3,3
CD4: reference plots
CD4-activated
TNFα/GM-CSF DP
IFNγ/IL2 DP
28,3
37,6
2,9
45,6
36,3
9,1
8,9
40,4
59,6
CD137-BV421
CD154-PE-CF594
TNFα-BV605
GM-CSF-APC
IFNγ-AF700
IL2-PE-Cy7
CCL4-PE
I
CD8
CD8-activated
5,6
CD8: reference plots
5,9
1,2
0,2
65,2
30,4
20
80
J
medium
E2p1
E2p2
E2p3
E2p4
E6/E7
PHA
0,23
1,07
0,60
0,48
0,29
3,21
31,2
0,044
0,006
0,002
0,32
0,40
0,054
0,28
0,076
0,008
0,21
0,038
0,001
0,078
0,039
0,009
1,15
1,33
0,12
11,0
1,19
0,46

Figure S1.

Figure S1. HPV16 E2-specific CD4+ and CD8+ T-cells can be found in TIL of CxCa patients, yet at lower frequency as E6 and E7-specific T-cells (related to Figure 1 and 2). E2 reactivity was determined in 9 CxCa patients with evident E6/E7 reactivity by manual gating. (A, D) Bar graph depicting total E2 and E6/E7-reactive CD4+ T-cells (A) and CD8+ T-cells (D). A response is depicted E2- or E6/E7-reactive when the percentage of cytokine-producing cells exceeds two times the medium control and has at least 10 positive spots in a gate. The total frequency of E2 and E6/E7-reactive T-cells is calculated as the SUM of all possible cytokine combinations as depicted in supplemental table I. (B, E) Bar graph depicting the frequency of the E2- or E6/E7-reactive CD4+ T-cells (B) and CD8+ T-cells (E) within E2 peptide pools 1 to 4. (C, F) Bar graph depicting frequency of E2- (left) and E6/E7-reactive (right) CD4+ T-cells (C) and CD8+ T-cells (F) in CxCa and OPSCC patients. To quantify polyfunctionality, HPV16-reactive CD4+ and CD8+ T-cells were subsequently subjected to manual gating. The gating strategy for polyfunctional T-cells is depicted for a representative OPSCC sample. (G) Cells were gated for singlets, live, CD3, CD4 and CD8 expression. (H, I) Activated CD4 (H) and CD8 (I) cells were selected by CD137 and/or CD154 expression and further analyzed for TNFα, GM-CSF, IFNγ, IL2 and CCL4 expression. Gates for cytokines were set on total CD4 and CD8 T-cells (reference plots). A gating example for sequential gating of the 5 cytokines is depicted for CD4 (H) and CD8 (I) cells. All possible cytokine combinations are given in supplemental Table I. (J) CD137/CD154 expression (top) and TNFα/GM-CSF expression (bottom) is depicted for CD4+ T cells in response to medium, E2p1-4 and E6/E7-loaded target cells and PHA. Red asterisk indicates a positive response, as defined by a frequency of at least two times the medium control.

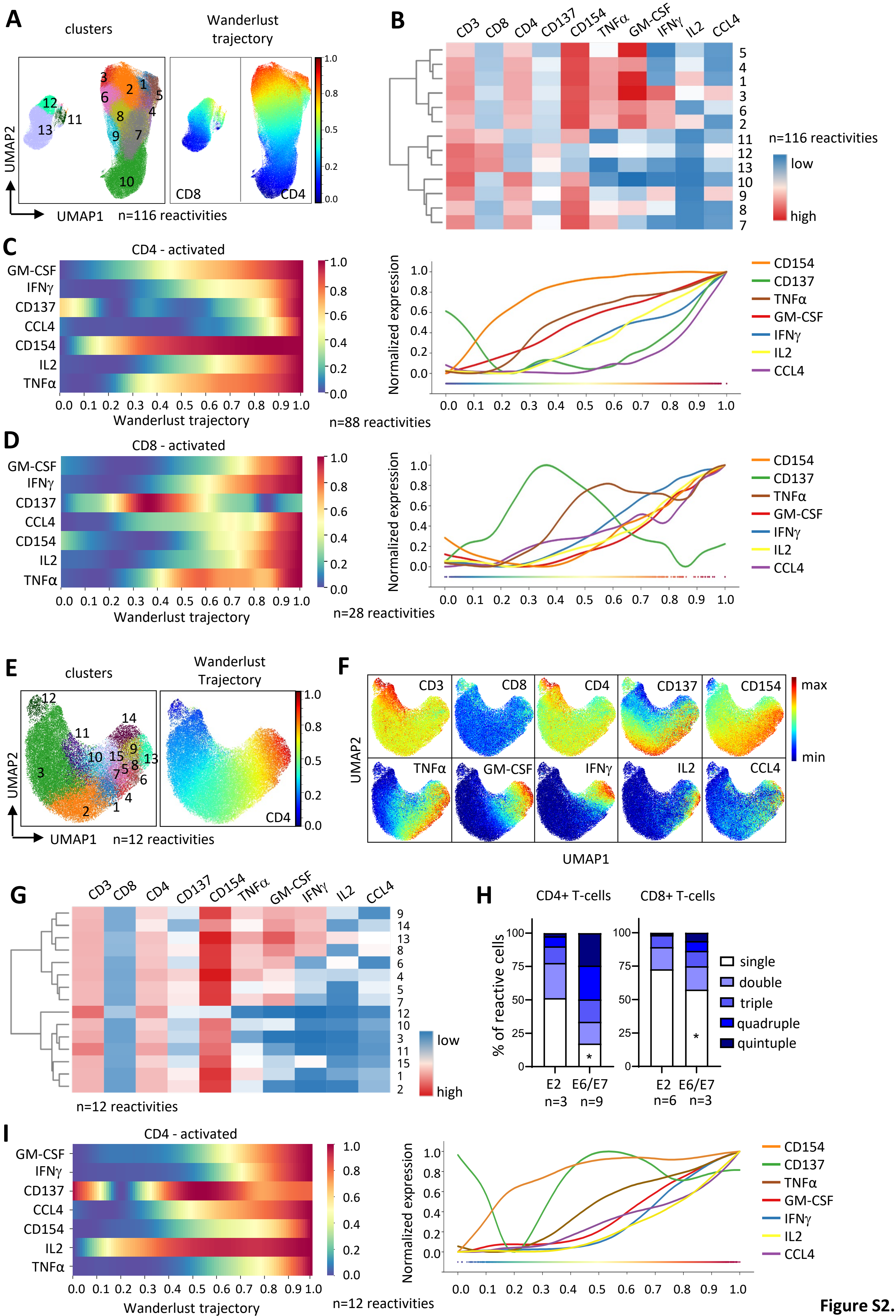
A
clusters
Wanderlust trajectory
UMAP2
UMAP1
n=116 reactivities
CD8
CD4
B
CD3 CD8 CD4 CD137 CD154 TNFα GM-CSF IFNγ IL2 CCL4
n=116 reactivities
low
high
C
CD4 - activated
GM-CSF
IFNγ
CD137
CCL4
CD154
IL2
TNFα
Wanderlust trajectory
n=88 reactivities
Normalized expression
D
CD8 - activated
n=28 reactivities
E
clusters
Wanderlust Trajectory
n=12 reactivities
F
max
min
UMAP2
UMAP1
G
n=12 reactivities
H
CD4+ T-cells
CD8+ T-cells
% of reactive cells
single
double
triple
quadruple
quintuple
E2 n=3
E6/E7 n=9
E2 n=6
E6/E7 n=3
I
CD4 - activated
n=12 reactivities

Figure S2.

Figure S2. HPV16 E2, E6 and E7-specific CD8+ and CD4+ T-cells in OPSCC and CxCa are polyfunctional (related to Figure 2). High-dimensional single cell data analysis of 116 activated CD4+ and activated CD8+ T-cell populations from OPSCC (A-D) and 12 activated CD4+ and activated CD8+ T-cell populations from CxCa (E-I) using OMIQ software. Dimensionality reduction using Uniform Manifold Approximation and Proximity (UMAP) analysis, FlowSOM consensus metaclustering and wanderlust trajectory analysis was performed on 245,072 activated CD4+ T-cells and 47,317 activated CD8+ T-cells for OPSCC and on 35,875 activated CD4+ T-cells for CxCa. (A) Overlay of 13 FlowSOM clusters (left) and Wanderlust trajectory (right; blue: begin and red: end) for the activated CD4+ and CD8+ T-cell populations in OPSCC plotted on the UMAP. (B) Hierarchically clustered heatmap of the FlowSOM clusters for OPSCC. Marker expression is shown as the z-score of median signal intensity per channel. (C, D) Heatmap plot (left) and line plot (right) displaying Wanderlust trajectory progression for 88 CD4-activated (C) and 28 CD8-activated (D) T-cell populations detected in OPSCC. (E) Overlay of 15 FlowSOM clusters (left) and wanderlust trajectory (right; color coding: blue: begin and red: end) plotted on the UMAP for the activated CD4+ T-cell populations in CxCa. (F) Expression intensity of the cell surface and cytokine markers in the CD4-activated T cells in CxCa plotted on the UMAP plot with blue as low and red as high expression. (G) Hierarchically clustered heatmap of phenotypes of the identified FlowSOM clusters is depicted. The indicated marker expression is shown as z-score of median signal intensity per channel. Blue: low expression, red: high expression. (H) Bar graphs depicting the percentage of single, double, triple, quadruple and quintuple cytokine-producing CD4+ T-cells (left) and CD8+ T-cells (right) for the total E2 and E6/E7-reactive T-cell populations in CxCa. (I) Heatmap plot (left) and line plot (right) displaying wanderlust trajectory progression for the markers CD137, CD154, TNFα, GM-CSF, IFNγ, IL-2 and CCL4 is depicted for CD4-activated T-cell populations detected in CxCa.

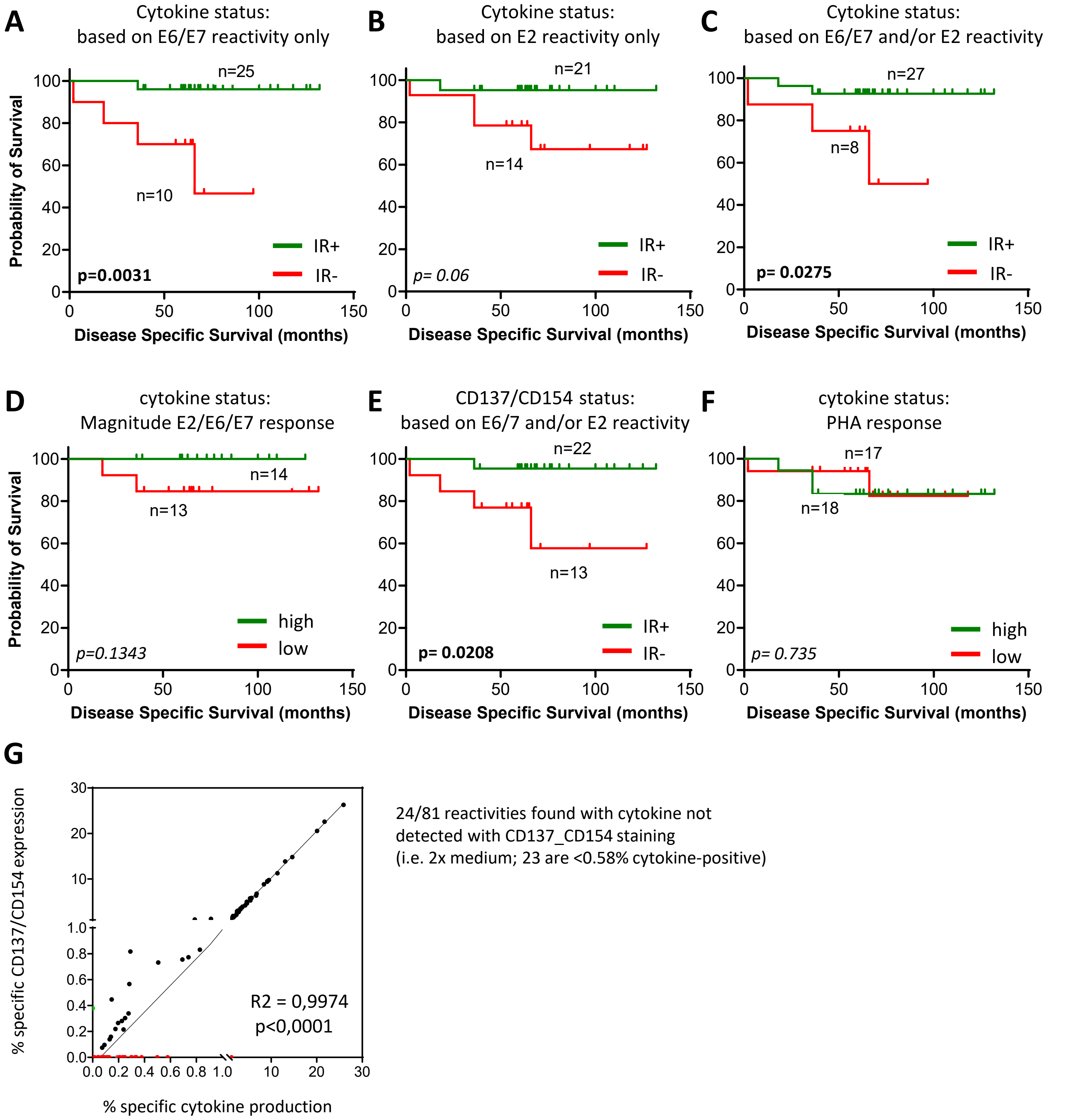

Figure S3. OPSCC patients with an immune response against HPV16 display superior survival (related to Figure 3). Kaplan-Meier survival curves of (A-C**)** 35 HPV16+ OPSCC patients who were analyzed by ICS. Patients were grouped based on the detection of an intratumoral HPV16 E2/E6/E7-specific T-cell response (i.e. immune response-positive (IR+; in green) or no detectable HPV16 E2/E6/E7-specific immune response (i.e. IR-negative (IR- in red)). IR status is given based on (A) detectable E6/E7-specific cytokine-producing T cells only, (B) E2-specific cytokine-producing T cells only and (C) E6/E7 and/or E2-specific cytokine-producing T cells for all patients. Kaplan-Meier survival curve of (D) 27 HPV16+IR+ OPSCC patients and (E, F) of 35 HPV16+ OPSCC patients who were analyzed by ICS. Patients were grouped based on (D) the detection of a high (i.e. > median (in green)) or low (i.e. < median (in red)) level of intratumoral total E2/E6/E7-specific cytokine-producing CD4+ T cells, (E) the detection of an intratumoral HPV16 E2/E6/E7-specific T-cell response (IR+; in green) or no detectable HPV16 E2/E6/E7-specific immune response (IR- in red) by CD137/CD154 expression and (F) the detection of a high (i.e. > median (in green)) or low (i.e. < median (in red)) level of intratumoral PHA-specific cytokine-producing CD4+ T cells.

(G) Correlation analysis between % specific CD137/CD154 expression and % specific cytokine production of E2 and E6/E7-specific CD4+ T cell populations for 81 different E2 and E6/E7-specific CD4+ T cell populations identified.

Table S1. Polyfunctional populations (Related to Figure 2 and STAR methods).

Population	How many cytokines
TNFa+GMCSF-IFNg+IL2-CCL4-	2
TNFa+GMCSF-IFNg+IL2-CCL4+	3
TNFa+GMCSF-IFNg+IL2+CCL4-	3
TNFa+GMCSF-IFNg+IL2+CCL4+	4
TNFa+GMCSF-IFNg-IL2+CCL4-	2
TNFa+GMCSF-IFNg-IL2+CCL4+	3
TNFa+GMCSF-IFNg-IL2-CCL4-	1
TNFa+GMCSF-IFNg-IL2-CCL4+	2
TNFa+GMCSF+IFNg+IL2-CCL4-	3
TNFa+GMCSF+IFNg+IL2-CCL4+	4
TNFa+GMCSF+IFNg+IL2+CCL4-	4
TNFa+GMCSF+IFNg+IL2+CCL4+	5
TNFa+GMCSF+IFNg-IL2+CCL4-	3
TNFa+GMCSF+IFNg-IL2+CCL4+	4
TNFa+GMCSF+IFNg-IL2-CCL4-	2
TNFa+GMCSF+IFNg-IL2-CCL4+	3
TNFa-GMCSF+IFNg+IL2-CCL4-	2
TNFa-GMCSF+IFNg+IL2-CCL4+	3
TNFa-GMCSF+IFNg+IL2+CCL4-	3
TNFa-GMCSF+IFNg+IL2+CCL4+	4
TNFa-GMCSF+IFNg-IL2+CCL4-	2
TNFa-GMCSF+IFNg-IL2+CCL4+	3
TNFa-GMCSF+IFNg-IL2-CCL4-	1
TNFa-GMCSF+IFNg-IL2-CCL4+	2
TNFa-GMCSF-IFNg+IL2-CCL4-	1
TNFa-GMCSF-IFNg+IL2-CCL4+	2
TNFa-GMCSF-IFNg+IL2+CCL4-	2
TNFa-GMCSF-IFNg+IL2+CCL4+	3
TNFa-GMCSF-IFNg-IL2+CCL4-	1
TNFa-GMCSF-IFNg-IL2+CCL4+	2
TNFa-GMCSF-IFNg-IL2-CCL4-	0
TNFa-GMCSF-IFNg-IL2-CCL4+	1

Table S2. Patient characteristics (Related to STAR methods).

**Patient ID** *	**Sex**	**Age****	**Tumor location**	**Received treatment**
**H35**	M	48	Tonsillar Fossa	RT
**H68**	F	64	Tonsil	RT
**H71**	F	62	Tongue base	RT
**H81**	M	48	Tonsillar Fossa	S+RT+CT
**H93**	M	57	Tongue base	S+RT
**H97**	M	70	Posterior wall	RT
**H103**	M	66	Tonsil	RT
**H104**	F	78	Tongue base	S+RT
**H133**	M	53	Tonsillar Fossa	RT
**H136**	F	47	Tonsil	CT+RT
**H138**	M	74	Tongue base	RT
**H139**	M	57	Tongue base	CT+RT
**H147**	M	57	Tonsil	CT+RT
**H148**	F	64	Tonsil	S+RT
**H149**	F	47	Tonsil	S+RT
**H150**	M	59	Tonsil	S+RT
**H160**	M	69	Tongue base	CT+RT
**H170**	M	60	Vallecula	S+RT
**H173**	F	60	Tongue base	CT+RT
**H180**	M	59	Tonsil	RT
**H182**	M	66	Tongue base	CT
**H185**	M	58	Tongue base	RT
**H188**	F	56	Tonsil	CT+RT
**H191**	M	39	Tonsil	RT
**H193**	M	67	Tongue base	RT
**H195**	F	62	Tongue base	CT+RT
**H196**	M	73	Tongue base	CT+RT+CX
**H202**	M	81	Tongue base	RT
**H206**	M	58	Tonsil	RT
**H208**	M	60	Tongue base	CT+RT
**H209**	M	70	Tonsil	CT+RT
**H211**	M	62	Tongue base	CT+RT
**H218**	M	58	Tongue base	RT
**H225**	M	67	Tongue base	RT
**H227**	F	44	Tonsil	CT+RT
**C176**	F	45	Cervix	S
**C178**	F	40	Cervix	S
**C194**	F	67	Cervix	S+RT
**C267**	F	49	Cervix	S+RT+CT
**C334**	F	42	Cervix	S+RT
**C446**	F	28	Cervix	S+RT
**C469**	F	42	Cervix	S
**C1016**	F	68	Cervix	S+RT
**C1051**	F	39	Cervix	S

* 'H' indicates OPSCC patients included in the P07-112 head and neck cancer study and "C" indicated CxCa patients included in the CIRCLE study.

** Age at diagnosis and sampling of tumor tissue pre-therapy

CT: chemotherapy; CX: cetuximab; F: female; M: male; RT: radiotherapy; S: surgical resection
